# Roller pump reverse flow-directed dynamic occlusiveness: towards precision in perfusion

**DOI:** 10.1093/icvts/ivae026

**Published:** 2024-02-24

**Authors:** Ignazio Condello, Enrico Squiccimarro

**Affiliations:** Department of Cardiac Surgery, Anthea Hospital, GVM Care & Research, Bari, Italy; Division of Cardiac Surgery, Department of Medical and Surgical Sciences, University of Foggia, Foggia, Italy

**Keywords:** Centrifugal pump, Roller pump, Vacuum-assisted venous drainage, Cardiopulmonary bypass

During CPB, mechanical pumps move blood over non-biological surfaces, including the oxygenator, in a non-laminar fashion. In this context, we read with great interest the article: ‘Effect of cardiopulmonary bypass on coagulation factors II, VII and X in a primate model: an exploratory pilot study’ by Shimoda *et al.* Cardiopulmonary bypass (CPB) is an underlying factor for coagulopathy following cardiac surgeries due to major blood loss that often necessitates allogenic blood transfusions and reoperations. Two major mechanisms are theorized to contribute to CPB-mediated coagulopathy, namely consumptive coagulopathy and haemodilution due to priming solution [1]. These complexities may stem from several unresolved dilemmas in the field. Among others, the question arises: which is the best pump? The development of centrifugal pumps (CP) was an undeniable breakthrough in the perfusion community. However, the evidence comparing roller (RP) and CP is far from conclusive [2]. During CPB is the mismatch between the calculated and measured flow (via ultrasonic flowmeter) of the master RP, with the first usually overestimating the actual flow. A significant variable, possibly impacting this phenomenon, is the degree of pump occlusion. Despite its known association with haemolysis, inadequate occlusion (both tight occlusive and non-occlusive settings) remains an unaddressed issue, with no international standards for adjusting occlusiveness [3]. The actual flow delivered by the master RP is a function of several aspects of CPB management, including device-related, disposable-related, patient-related, or procedure-related factors, such as pump model, tubing material (silicone or PVC) and diameter, temperature, blood viscosity, oxygenator model, design and pressure drop, the use of vacuum-assisted venous drainage and the choice between continuous or pulsatile flow. These hurdles undermine the integration of RP within the concept of ‘precision perfusion’, potentially paving the way for further adoption of CP, even in complex scenarios like combined use of vacuum-assisted venous drainage. While not opposing the use of CP, nor blindly inclining towards RP, we acknowledge the potential implications of optimized RP design and functionality. We present the concept of a ‘reverse flowdirected RP dynamic occlusiveness’, involving continuous monitoring of reverse flow at the pump inlet (directly related to the mismatch between calculated and measured flow) and dynamic adjustment of RP occlusion via digital readouts, without interrupting CPB flow. This approach, intended to be integrated into algorithms, is not designed to reshape the work of the perfusionist, who would not be required to master new tasks beyond the established and familiar routine. However, the concept of ‘reverse flow-directed RP dynamic occlusiveness’ could open new horizons towards embracing precision medicine practices in perfusion.
